# Components and effectiveness of patient navigation programmes to increase participation to breast, cervical and colorectal cancer screening: A systematic review

**DOI:** 10.1002/cam4.6050

**Published:** 2023-05-28

**Authors:** Isabel Mosquera, Adam Todd, Mirza Balaj, Li Zhang, Sara Benitez Majano, Keitly Mensah, Terje Andreas Eikemo, Partha Basu, Andre L. Carvalho

**Affiliations:** ^1^ Early Detection, Prevention & Infections Branch, International Agency for Research on Cancer Lyon France; ^2^ School of Pharmacy Newcastle University, Newcastle upon Tyne UK; ^3^ Centre for Global Health Inequalities Research (CHAIN), Department of Sociology and Political Science Norwegian University of Science and Technology (NTNU) Trondheim Norway; ^4^ Noncommunicable Diseases, Violence and Injuries Prevention Unit, Pan American Health Organization Washington DC USA; ^5^ Inequalities in Cancer Outcomes Network, London School of Hygiene and Tropical Medicine London UK

**Keywords:** breast cancer, cervical cancer, colorectal cancer, components, effectiveness, patient navigation, screening

## Abstract

**Background:**

Inequalities in cancer incidence and mortality can be partly explained by unequal access to high‐quality health services, including cancer screening. Several interventions have been described to increase access to cancer screening, among them patient navigation (PN), a barrier‐focused intervention. This systematic review aimed to identify the reported components of PN and to assess the effectiveness of PN to promote breast, cervical and colorectal cancer screening.

**Methods:**

We searched Embase, PubMed and Web of Science Core Collection databases. The components of PN programmes were identified, including the types of barriers addressed by navigators. The percentage change in screening participation was calculated.

**Results:**

The 44 studies included were mainly on colorectal cancer and were conducted in the USA. All described their goals and community characteristics, and the majority reported the setting (97.7%), monitoring and evaluation (97.7%), navigator background and qualifications (81.4%) and training (79.1%). Supervision was only referred to in 16 studies (36.4%). Programmes addressed mainly barriers at the educational (63.6%) and health system level (61.4%), while only 25.0% reported providing social and emotional support. PN increased cancer screening participation when compared with usual care (0.4% to 250.6% higher) and educational interventions (3.3% to 3558.0% higher).

**Conclusion:**

Patient navigation programmes are effective at increasing participation to breast, cervical and colorectal cancer screening. A standardized reporting of the components of PN programmes would allow their replication and a better measure of their impact. Understanding the local context and needs is essential to design a successful PN programme.

## 1 INTRODUCTION

In the last decades cancer incidence and mortality have been increasing worldwide, across high‐ and low and middle‐income countries (LMIC).^1^ One approach that has been shown to reduce breast, cervical and colorectal cancer‐related mortality at a population level, is cancer screening.^2^, ^3^, ^4^ For cancer screening to be as effective as possible, it is important that screening programmes reach high coverage of the target population. This is achieved with screening programmes easily accessible and available to everyone – regardless of their socioeconomic position.

Several interventions have been described to promote equitable cancer screening and reduce structural barriers related to access (e.g., the use of mobile units), but one approach that is gaining significant interest is patient navigation. In 1990 Freeman developed patient navigation to assist low‐income women in USA to overcome barriers to follow‐up after an abnormal breast cancer screening result.^5^ As patient navigation began to take shape, it was implemented in the screening of other cancer sites, at different levels of the cancer screening continuum and for other socially disadvantaged groups. Therefore, a patient navigation intervention is by default designed to improve access among hard‐to‐reach populations. Moreover, the patient navigation approach is focused on supporting people to overcome barriers and has the following characteristics: (i) occurs within a specific cancer care event (e.g., one‐time screening), (ii) follows the individual until a specific endpoint is reached (e.g., a definitive diagnosis), (iii) targets the health services needed to achieve the endpoint (e.g., screening and/or diagnostic care), (iv) addresses barriers at a health system, individual, educational, and/or social and emotional level, and (v) aims to reduce delays in cancer care access and uptake.^6^

As the evidence for patient navigation developed, DeGroff et al. (2014) outlined 10 key components that should be considered when designing a patient navigation programme: (1) identification of a theoretical framework and establishment of programme goals, (2) specification of the community characteristics, (3) determination of the point(s) of intervention in the cancer care continuum, (4) establishment of the setting where navigation is provided, (5) description of the services offered and the patient navigator responsibilities, (6) determination of the background and qualifications of navigators, (7) selection of the communication method between individuals and navigators, (8) design of the patient navigator training, (9) establishment of the supervision of navigators, and (10) evaluation of patient navigation.^7^

Previous systematic reviews have described the positive impact that patient navigation interventions have on improving screening participation for breast, cervical and colorectal cancer, although it is acknowledged most of these studies have been conducted in the USA. The literature has reported increased participation in patient navigation programmes when compared to control (as usual care) as well as other types of interventions.^6^, ^8^, ^9^ Although the previous reviews have been helpful to extend our understanding in this field and summarize a complex evidence base, the definition used by the studies to conceptualize a patient navigation intervention is wide‐ranging and varied. This is challenging, given interventions might be based on the concept of patient navigation, but may not necessarily use this term to describe them. To the best of our knowledge, a comprehensive review on patient navigation programmes using a framework guided by the key components outlined by DeGroff et al. has not been undertaken. This work sought to address this gap by identifying the reported components of patient navigation to consider when conceptualizing patient navigation, and by assessing the effectiveness of patient navigation programmes to promote breast, cervical and colorectal cancer screening.

## 2 METHODS

A systematic search of the literature was conducted in Embase, PubMed and Web of Science Core Collection databases in March 2020 and then updated (January 2021). The search was limited to papers published since 2000 (as previous literature was considered not relevant to our purpose) without language restriction. The search strategies combined Medical Subject Headings and free text terms regarding screening, breast, cervical and colorectal cancer, interventions, participation and social inequalities. As an example, the search strategy used in Web of Science Core Collection is presented in Appendix 1.

The population considered was all people eligible for breast, cervical or colorectal cancer screening as defined by the eligibility criteria for that screening. The screening methods considered were those recognized and validated in IARC (International Agency for Research on Cancer) handbooks.^10^, ^11^, ^12^ Interventions were patient navigation programmes that aimed to increase access to cancer screening. Although we did not require studies to identify their intervention as patient navigation, we included only those where the intervention was individualized and ready to address some type of barrier, specifying it or not. The outcome was participation in cancer screening comparing patient navigation against usual care or other interventions. Screening participation could be extracted from health service records or as a self‐report. Included study designs were controlled trials, cohort studies, repeat cross‐sectional studies, case–control studies, before‐after studies and after only studies. Studies that were not original, reported several interventions or interventions targeting only populations at high risk of developing cancer (e.g., genetic/familial disorders) were excluded from the review.

Inclusion and exclusion criteria were piloted in 100 references before their application to the whole set of identified references, discussing any discrepancies until a consensus was reached among researchers. Two researchers (IM and LZ) independently assessed titles and/or abstracts of the identified references using Covidence software, with a third (AC) in case of discrepancy. Two researchers (IM and LZ) read 40% of the full‐text references and Cohen's Kappa statistic was used to measure the interrater reliability (IRR). As Kappa was higher than 0.7, the first reviewer assessed the remaining references.

After the selection of the included studies, the following information was extracted for each study and included in an Excel spreadsheet: period of analyzed data, country, cancer site, components of patient navigation^7^ – ‘theoretical framework’, ‘programme goals’, ‘community characteristics’, ‘point of intervention’, ‘services provided’, ‘communication method’, ‘navigator background and qualification’, ‘training’, ‘supervision’, and ‘monitoring and evaluation (other than screening participation)’ – participants – number, age group and percentage of females when applicable – measure of socioeconomic position of the population included and measure of socioeconomic position in the analysis if applicable, study design, comparison, screening method and main findings – outcome, including baseline characteristics.

The components of patient navigation examined were 14 instead of the 10 described by DeGroff et al.,^7^ as ‘theoretical framework and programme goals’ were broken down into two components, and ‘services provided’ by patient navigators were divided into four components based on the categories of barriers addressed. These categories were: (a) health system barriers (including scheduling appointments, paperwork and patient‐provider communication), (b) individual barriers to cancer screening (lack of transportation, financial and insurance barriers, lack of childcare or language translation, low health literacy or low literacy), (c) educational barriers related to cancer and screening and (d) social and emotional barriers.^6^

The data extraction was carried out by one reviewer (IM or SBM) and revised and completed by a second reviewer (SBM or IM). Disagreements were resolved by open discussion. If a consensus was not reached, a third reviewer was consulted (AC), and the majority decision was followed. In studies providing screening participation rate, the percentage increase in participation following the intervention was calculated.

The methodological quality of the included studies was assessed independently by two reviewers (IM and KM) through the application of the study quality assessment tools of the National Heart, Lung and Blood Institute for quantitative studies.^13^ Studies were classified into three quality categories (good, fair and poor) based on criteria regarding study design, including randomization, blinding, drop‐out and outcome measures, among others. Discrepancies were reconciled through discussion.

A meta‐analysis of the studies was planned, but their heterogeneity hindered this, and findings were synthesized by means of a narrative synthesis.^14^ The systematic review was registered with PROSPERO (CRD42020193657).

## 3 RESULTS

**FIGURE 1:**
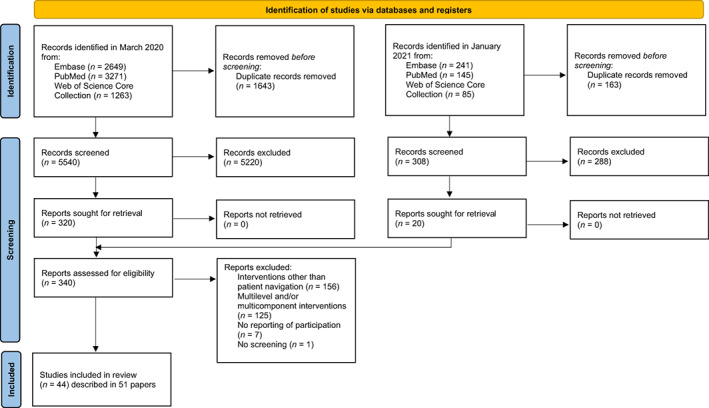
Study selection flow diagram.

The initial systematic search identified 5540 references to screen after taking out duplicates, and the updated search found an additional 308 references. After the application of inclusion and exclusion criteria, 5508 references were deemed not relevant for the topic of interest, resulting in 340 references selected to be read full text. Finally, 51 references on patient navigation were included (Figure 1), corresponding to 44 studies.

Studies on ‘patient navigation’ rarely defined this term. Most studies were randomized controlled trials and assessed colorectal cancer screening participation (Appendix 2).^15^, ^16^, ^17^, ^18^, ^19^, ^20^, ^21^, ^22^, ^23^, ^24^, ^25^, ^26^, ^27^, ^28^, ^29^, ^30^, ^31^, ^32^, ^33^, ^34^, ^35^, ^36^, ^37^, ^38^, ^39^, ^40^, ^41^, ^42^, ^43^, ^44^, ^45^, ^46^, ^47^, ^48^, ^49^, ^50^, ^51^, ^52^, ^53^, ^54^, ^55^, ^56^, ^57^, ^58^, ^59^, ^60^, ^61^, ^62^, ^63^, ^64^ Participant numbers ranged from 49^49^ to 28,929.^24^, ^25^ The most common measure of socioeconomic position was ethnicity/race,^16^, ^17^, ^22^, ^26^, ^27^, ^28^, ^32^, ^38^, ^40^, ^41^, ^44^, ^47^, ^48^, ^50^, ^51^, ^52^, ^58^, ^59^, ^62^, ^64^ followed by income,^15^, ^20^, ^31^, ^39^, ^48^, ^49^, ^51^, ^52^, ^59^, ^63^ and geographic area. ^8^, ^18^, ^19^, ^24^, ^25^, ^31^, ^32^, ^45^, ^46^, ^59^ Other measures used were health insurance,^15^, ^30^, ^41^, ^48^, ^52^, ^59^, ^64^ primary language,^21^, ^29^, ^30^, ^39^, ^40^, ^59^, ^64^ language preference,^61^ education,^52^ health literacy,^15^, ^18^, ^19^, ^26^, ^45^, ^46^, ^50^ employment,^49^ material deprivation^24^, ^25^ or socioeconomic status.^32^

The screening methods evaluated were mammography^44^, ^45^, ^46^, ^47^, ^48^, ^49^, ^50^, ^51^, ^52^, ^53^ and clinical breast examination^47^ for breast cancer, cytology^8^, ^54^, ^56^, ^57^, ^58^ and HPV self‐sampling^56^, ^57^ for cervical cancer, and fecal occult blood test (FOBT),^15^, ^16^, ^17^, ^18^, ^19^, ^21^, ^22^, ^23^, ^24^, ^25^, ^26^, ^29^, ^30^, ^32^, ^34^, ^35^, ^36^, ^37^, ^38^, ^39^, ^40^, ^42^, ^43^ fecal immunochemical test (FIT),^31^, ^41^ colonoscopy,^17^, ^20^, ^21^, ^22^, ^23^, ^26^, ^27^, ^28^, ^29^, ^30^, ^31^, ^32^, ^34^, ^35^, ^36^, ^37^, ^38^, ^39^, ^40^, ^42^, ^43^ sigmoidoscopy,^21^, ^22^, ^23^, ^26^, ^29^, ^30^, ^32^, ^33^, ^34^, ^35^, ^36^, ^37^, ^39^, ^40^, ^43^ barium enema^21^, ^29^, ^30^, ^32^, ^34^, ^35^, ^36^, ^37^, ^38^, ^39^, ^40^ and virtual colonoscopy^32^ for colorectal cancer screening. Studies were carried out mainly in the USA, although studies from other geographic areas (Australia,^54^ Canada,^58^ France,^24^, ^25^ UK^56^, ^57^ and Zambia^55^) were also identified.

The quality of the included studies was mainly rated as poor (Appendix 2). The most frequent shortcomings were a lack of, or not reporting the justification of the sample size, a concealed allocation and a drop out higher than 20%.

### 3.1 Components of patient navigation

Most studies (*n* = 37, 84.1%) had at least 9 out of 14 components of patient navigation. All studies described the goals of the navigation programme, community characteristics and point of intervention (Table 1, Appendices 3 and 4). Programme goals were generally to increase screening participation among disadvantaged populations.

**TABLE 1:** Components of patient navigation programmes described in the included studies.

Components of patient navigation programme	Overall *N* (%)
Programme goals	44 (100)
Community characteristics	44 (100)
Point of intervention	44 (100)
Setting	43 (97.7)
Monitoring and evaluation (other than screening participation)	43 (97.7)
Communication	42 (95.5)
Background and qualifications	35 (81.4)^a^
Training	34 (79.1)^a^
Address educational barriers	28 (63.6)
Address health system barriers	27 (61.4)
Theoretical framework	21 (47.7)
Address individual barriers	21 (47.7)
Supervision	16 (36.4)
Address social and emotional barriers	11 (25.0)

^a^ Excludes one study where this component was not applicable.

Studies were conducted predominantly among hard‐to‐reach populations, although there were studies with primary care patients,^23^, ^31^, ^34^, ^35^, ^36^, ^42^, ^51^ general target population,^24^, ^25^, ^55^, ^56^, ^57^ and with population living in an area with relatively unrestricted accessibility to resources.^54^ The majority of the studies targeted populations not up to date with screening.^8^, ^17^, ^18^, ^19^, ^21^, ^22^, ^23^, ^27^, ^29^, ^30^, ^34^, ^35^, ^36^, ^37^, ^38^, ^39^, ^40^, ^42^, ^44^, ^45^, ^46^, ^47^, ^56^, ^57^, ^58^, ^61^, ^63^, ^64^

Patient navigators were located mainly at a primary care or community level,^8^, ^15^, ^16^, ^17^, ^18^, ^19^, ^21^, ^22^, ^23^, ^26^, ^27^, ^30^, ^31^, ^32^, ^33^, ^34^, ^35^, ^36^, ^37^, ^38^, ^39^, ^40^, ^41^, ^42^, ^43^, ^44^, ^45^, ^46^, ^47^, ^48^, ^50^, ^51^, ^55^, ^56^, ^57^, ^58^, ^60^, ^61^, ^63^, ^64^ and communicated with participants through phone calls.^8^, ^15^, ^16^, ^17^, ^18^, ^19^, ^20^, ^21^, ^22^, ^23^, ^24^, ^25^, ^27^, ^28^, ^29^, ^30^, ^31^, ^32^, ^33^, ^34^, ^36^, ^37^, ^38^, ^39^, ^41^, ^42^, ^43^, ^44^, ^45^, ^46^, ^47^, ^48^, ^50^, ^51^, ^52^, ^53^, ^54^, ^55^, ^56^, ^58^, ^59^, ^61^, ^62^, ^63^, ^64^ In some cases they met in person,^8^, ^18^, ^20^, ^24^, ^25^, ^28^, ^39^, ^42^, ^44^, ^45^, ^46^, ^47^, ^49^, ^50^, ^51^, ^55^, ^58^, ^62^ and/or sent lette rs,^22^, ^24^, ^25^, ^50^, ^51^, ^52^, ^56^, ^61^, ^62^ e‐mails^20^, ^23^, ^56^, ^59^ or text messages.^56^, ^59^ Regarding the services they provided, 27 studies (61.4%) described them addressing health system barriers, as scheduling appointments,^18^, ^20^, ^23^, ^30^, ^32^, ^33^, ^34^, ^39^, ^40^, ^42^, ^44^, ^47^, ^50^, ^53^, ^56^, ^58^, ^61^, ^62^ sending reminders,^15^, ^16^, ^18^, ^19^, ^20^, ^21^, ^27^, ^32^, ^34^, ^35^, ^37^, ^38^, ^39^, ^40^, ^42^, ^45^, ^46^, ^56^, ^61^, ^62^, ^63^, ^64^ completing paperwork,^62^ or communicating or supporting communication with providers.^20^, ^44^, ^50^, ^62^ In 21 studies (47.7%), they approached individual barriers, through assistance in transportation,^20^, ^27^, ^39^, ^40^, ^47^, ^53^, ^58^, ^62^, ^64^ escorting to appointments,^20^, ^30^, ^39^, ^40^, ^44^, ^49^, ^50^, ^55^, ^64^ or arranging the care of dependents during the appointment.^62^ Language^39^, ^40^, ^47^, ^52^, ^58^, ^63^ and financial barriers^30^, ^62^ were also addressed by navigators. Four studies (9.1%) did not specify what type of barriers they helped approach.^26^, ^43^, ^48^, ^59^ Navigators were reported to provide education in 28 studies (63.6%), but only 11 (25.0%) described social and emotional support.

The background and qualifications of the navigator were described in 35 studies (81.4%). Most of them referred the navigators' language, culture or ethnicity (generally because it being the same as that of the study participants),^8^, ^20^, ^21^, ^22^, ^27^, ^30^, ^39^, ^40^, ^44^, ^47^, ^50^, ^51^, ^52^, ^58^, ^59^, ^61^, ^63^, ^64^ while others indicated their educational level or occupation.^8^, ^15^, ^18^, ^19^, ^20^, ^22^, ^24^, ^25^, ^27^, ^29^, ^30^, ^32^, ^33^, ^37^, ^39^, ^40^, ^43^, ^47^, ^49^, ^57^, ^61^, ^62^ The occupation of the navigators included health educators,^30^, ^47^, ^56^, ^57^ nurses,^15^, ^18^, ^19^, ^23^, ^29^, ^30^, ^37^, ^41^, ^42^, ^45^ lay persons,^20^, ^27^, ^43^, ^51^, ^62^ specialist screening practitioners,^33^ community health workers,^30^ social workers^24^, ^25^ or community leaders.^60^ Patient navigators had reached college,^28^, ^29^, ^30^, ^39^, ^40^, ^61^, ^64^ obtained a master's degree^32^, ^53^ or bachelor's degree,^22^, ^27^, ^30^ or were first year medical students.^49^ Some studies reported their experience in community health outreach,^29^, ^30^ or telephone interventions.^32^, ^53^ One study used an interactive voice response (IVR) navigation.^54^

Training of navigators was reported in 34 studies (79.1%), mainly indicating the duration of training.^20^, ^21^, ^22^, ^24^, ^25^, ^27^, ^28^, ^29^, ^30^, ^39^, ^43^, ^44^, ^47^, ^49^, ^53^, ^60^, ^62^, ^63^ The shorter trainings were for prevention care managers (half a day^21^) and health centre outreach workers and interpreters (6 h).^39^ For lay navigators, reported duration of training was 2 days^20^ to 19 h plus a series of one‐on‐one structured role plays simulating a navigation encounter.^27^ The maximum duration referred was 80 h for navigators with a bachelor's degree in public health or a related field^22^ and in a study not specifying the navigator's background.^44^ Studies with nurse navigators did not clarify the length of training.

Most studies reported ad hoc training programmes, with few referring an already existing programme from an institution or a standardized national programme providing a certification.^20^, ^22^, ^26^, ^52^ Twelve studies described the contents of training,^18^, ^19^, ^21^, ^27^, ^28^, ^29^, ^30^, ^39^, ^44^, ^47^, ^50^, ^58^, ^60^ which included education on a selected cancer and its screening,^30^, ^39^, ^44^, ^49^, ^60^, ^64^ topics related to care (common patient barriers^28^ and how to address them,^51^ local community resources,^28^, ^44^ and appropriate follow up for abnormal results^60^), navigator roles^28^, ^60^ and responsibilities,^27^, ^28^ skills in communication (motivational interviewing techniques,^18^, ^19^, ^20^, ^30^, ^39^, ^42^, ^46^, ^64^ interview protocols,^53^ communication with clinicians^64^), and monitoring and evaluation (data management,^28^, ^44^, ^49^, ^64^ quality measures^28^). Seven studies reported the use of role‐playing as a learning strategy.^21^, ^29^, ^30^, ^43^, ^50^, ^53^, ^60^ Three studies had continuing education sessions,^22^, ^44^, ^62^ three included site visits to clinics^27^, ^43^, ^44^ and one the attendance to a colonoscopy.^24^ No study described training in confidentiality and privacy practices. The supervision of navigators was referred to in only 16 studies (36.4%), and when described the supervision was often achieved through regular meetings,^21^, ^25^, ^28^, ^29^, ^30^, ^43^, ^60^ or auditing navigator telephone calls.^29^, ^30^, ^48^, ^53^

Apart from screening participation, studies monitored and evaluated a wide variety of indicators related to the navigation process, more specifically on communication, barriers reported, navigation services provided and time. With regards to communication, indicators used were the number of study participants contacted,^21^, ^30^, ^33^, ^39^, ^51^, ^53^, ^63^ number of contacts^21^, ^22^, ^29^, ^30^, ^32^, ^43^, ^47^, ^52^, ^59^, ^63^ or duration of contact.^21^, ^24^, ^29^, ^30^, ^33^, ^43^, ^53^ Few studies stated the number of attempts patient navigators made to reach participants, those that did range from up to 3^48^, ^63^ to up to 12 attempts.^21^ In the main, studies did not report the average length of telephone calls,^21^, ^24^, ^43^, ^46^ with only two studies differentiating between initial and subsequent calls,^21^, ^24^ and two indicating the average total time spent with participants.^29^, ^30^

Studies described the most frequent barriers faced by study participants,^16^, ^18^, ^19^, ^22^, ^47^, ^51^, ^52^, ^59^ workload of navigators,^20^, ^21^, ^25^, ^28^, ^29^, ^34^, ^35^, ^38^, ^50^, ^62^ navigation services provided^25^, ^39^, ^52^, ^61^, ^62^ and time spent by activity.^37^ Several studies measured screening process or programme outcomes such as screen positivity^16^, ^44^ or number of cancer cases identified,^30^, ^39^, ^52^ attendance to follow‐up,^33^ predictors of participating in screening^8^, ^26^, ^27^, ^31^, ^49^, ^60^ and cost or cost‐effectiveness.^18^, ^19^, ^23^, ^28^, ^33^, ^37^, ^45^, ^46^, ^55^, ^56^, ^64^ Few studies measured knowledge and perception of screening,^38^, ^41^ satisfaction with navigation services^62^ or adverse events (after colonoscopy).^23^ One study assessed training outcomes, such as knowledge of navigator, level of abilities or satisfaction with training.^60^

Only 21 studies (47.7%) explicitly identified the theoretical framework used to inform the intervention. Most studies included behavioral frameworks, as the health belief model,^8^, ^15^, ^18^, ^19^, ^20^, ^36^, ^46^, ^47^ the transtheoretical model or stages of change model,^29^, ^30^, ^42^, ^47^, ^48^, ^60^ the theory of reasoned action^20^, ^53^ or the Precaution Adoption Process Model (PAPM),^32^, ^34^, ^35^, ^38^ the social cognitive theory.^8^, ^15^, ^16^, ^18^, ^19^, ^20^, ^36^, ^44^, ^46^, ^53^, ^59^, ^62^ and the Preventive Health Model (PHM).^34^, ^35^, ^38^ Other studies employed planning/evaluation frameworks, as PRECEDE (Predisposing, Reinforcing and Enabling Constructs in Educational Diagnosis and Evaluation)/PROCEED (Policy, Regulatory and Organizational Constructs in Educational and Environmental Development) planning model,^8^, ^53^ CEDIP (Clarify, Empathize, Disclose, Inform and Plan)/CEEP (Clarify, Empathize, Educate and Plan) model.^27^

The presence of components of patient navigation was quite similar across cancer sites. However, breast cancer screening studies reported more frequently training (*n* = 9, 100%) and addressing individual barriers (*n* = 6, 66.7%), while programmes for multiple cancer sites together were more likely to describe supervision (*n* = 3, 50%).

### 3.2 Effectiveness of patient navigation

Screening participation was reported based on medical record review in the majority of included studies, but some studies estimated participation based on self‐reporting only,^22^, ^26^, ^35^, ^43^, ^44^, ^50^, ^62^ while three used a combination of both methods.^8^, ^17^, ^34^ Screening participation was measured over a period ranging from 14 days^49^ to 30 months.^46^ Patient navigation increased screening participation for the three cancer sites regardless of the measure of socioeconomic measure considered (Table 2). It was compared with baseline/usual care,^8^, ^20^, ^21^, ^24^, ^25^, ^27^, ^28^, ^29^, ^30^, ^31^, ^33^, ^39^, ^40^, ^41^, ^42^, ^43^, ^47^, ^48^, ^54^, ^55^, ^56^, ^57^, ^58^, ^61^, ^62^, ^64^ enhanced usual care (frequently not described),^15^, ^18^, ^19^, ^36^, ^46^ or other interventions,^15^, ^16^, ^17^, ^18^, ^19^, ^22^, ^26^, ^31^, ^43^, ^44^, ^45^, ^46^, ^49^, ^50^, ^53^, ^56^, ^57^, ^58^, ^59^, ^63^ these being mainly educational interventions.^15^, ^18^, ^19^, ^22^, ^26^, ^44^, ^45^, ^46^, ^49^, ^50^, ^59^, ^63^ The proportion of participants assigned to patient navigation receiving the intervention ranged from 10.5%^33^ to 96.1%;^54^ few studies provided this information.

**TABLE 2:** Screening participation expressed as %, increase and/or odds ratio (OR), by cancer site.

First author, year	Comparison	Intervention	Screening participation (intervention vs comparison(s)) (%)	*p* value	OR (95% CI), intervention vs comparison(s)	*p* value
*Colorectal cancer*						
Arnold, 2016^41^	[1] enhanced version of usual care [2] health literacy‐informed educational intervention	[1] and nurse providing a health literacy‐informed education and telephone follow‐up using motivational interviewing	47.4% vs 34.2% [1] vs 59.6% [2] 44.4% vs 39.1% [1] vs 76.9[2] in limited health literacy 52.2% vs 26.7 [1] vs 53.9% [2] in adequate health literacy	0.21 after adjusting for age, race, sex, and health literacy 0.007 in limited health literacy 0.002 with adequate health literacy	1.35 (0.78–2.33) compared to enhanced care 0.75 (0.49–1.17) compared to education	0.28 0.20
Braun, 2005^19^	group education + small media + free FOBT + single telephone reminder call	group education + experience from Native Hawaiian CRC survivor + educational material + free FOBT + reminder calls (which included efforts to address personal emotions and barriers)	8% increase vs 16% increase	<0.05		
Cole, 2017^33^	[1] motivational interviewing for blood pressure control	[2]: [1] + patient navigation [3] Patient navigation	17.5% [3] vs 8.4% [1] vs 17.8% [2]		Compared to [1]: 2.32 (1.55–3.46) unadjusted; 2.43 (1.32–4.46) adjusted for education, hypertension awareness, self‐reported diabetes and health literacy	
Davis, 2013^43^ Davis, 2014^44^	[1] enhanced usual care [2] educational strategy	[3]: [2]+nurse support	Initial FOBT: 60.6% [3] vs 57.1% [2] Second FOBT: 59% vs 33% [2]	<0.001 0.017	Initial FOBT compared to [2]: 1.18 (0.97–1.42) Second FOBT compared to [2]: 1.46 (1.14–1.86)	0.09 0.002
DeGroff, 2017^42^	Usual care	Patient navigation	61.1% vs 53.2%	0.021	1.51 (1.12–2.03) 2.60 (1.64–4.13) Hispanics vs non‐Hispanic whites	0.007 ≤0.001
Dietrich, 2013^49^	Usual care	Prevention care management	36.7% vs 30.6% overall 32% vs 17.5% colonoscopy 14.7% vs 11.7% FOBT		1.32 (1.08–1.62) overall adjusted for age, comorbidities (diabetes, hypertension, and high cholesterol levels), visits within 18 months, insurance (Medicaid or Family Health Plus), and primary language, all at baseline 2.22 (1.62–3.05) colonoscopy 1.31 (0.87–1.95) FOBT	
Enard, 2015^28^	Mailing of educational materials	Patient navigation	43.7 vs 32.1% overall 35.6 vs 23.8 colonoscopy/FS	0.04 0.03	1.64 (1.02–2.62) 1.82 (1.11–3.00) adjusted for age, gender, education, and usual source of care provider status. Colonoscopy/FS: 1.77 (1.07–2.91) 1.90 (1.13–3.22) adjusted for age, gender, education, and usual source of care provider status FOBT: 1.57 (0.87–2.81) 1.79 (0.96–3.33) adjusted for age, gender, education, and usual source of care provider status	0.04 0.02 0.03 0.02 0.13 0.07
Green, 2013^57^	[1] Usual care [2]: [1] + automated care [3]: [2] + assisted care	[4]: [3] + navigated care	64.7% vs 57.5% [3] overall in both years 35.8% vs 30.5% [3] FOBT in both years			
Guillaume, 2017^16^ De Mil, 2018^17^	Usual postal reminder	Patient navigation	Screening population: 29 vs 27.5% 26.8 vs 25.6% (deprived strata) 24.2 vs 24.1 (urban deprived) 30 vs 27.2% (rural deprived) 31.6 vs 29.6% (affluent strata) 30.8 vs 30% (urban affluent) 32 vs 29.4% (rural affluent) Navigable population: 24.3 vs 21.1% 22.8 vs 20.2% (deprived strata) 21.5 vs 18.9% (urban deprived) 24.3 vs 21.3% (rural deprived) 26.0 vs 21.9% (affluent strata) 25.4 vs 22.1% (urban affluent) 26.6 vs 21.8% (rural affluent)	0.35 0.53 0.95 0.3 0.42 0.85 0.29 0.003 0.07 0.15 0.16 0.01 0.07 0.02	Screening population: 1.08 (0.99–1.18) 1.27 (1.12–1.44) (rural deprived vs urban deprived) 1.41 (1.22–1.63) (urban affluent vs urban deprived) 1.39 (1.24–1.56) (rural affluent vs urban deprived) Navigable population: 1.19 (1.10–1.29) 1.17 (1.05–1.31) (rural deprived vs urban deprived) 1.25 (1.11–1.41) (urban affluent vs urban deprived) 1.26 (1.13–1.40) (rural affluent vs urban deprived)	0.060 <0.001 <0.001 <0.001
Horne, 2015^30^	Printed educational materials	Patient navigation	94% vs 91% Among participants not up to date at baseline: 72.5% vs 58.6%	0.04 0.008	1.56 (1.08–2.25) Adequate health literacy compared to low health literacy: 1.11 (0.76–1.63) 70–74 years compared to 65–69 years: 0.98 (0.67–1.42)	0.02 0.57 0.90
Jandorf, 2013^23^	[1] Standard of care: patient navigation	[2] Peer‐patient navigation [3] Propatient navigation	74.0% [2] vs 76.4% [3] vs 80.4% [1]	0.648		
Kim, 2018^35^	No navigation	Patient navigation	85.1 vs 73.4%	<0.05		
Lasser, 2009^48^	Usual care	Patient navigation	31.2 vs 8.9% overall 17.2 vs 7.8% FOBT 14.0 vs 1.1% colonoscopy	0.0002 overall		
Lasser, 2011^47^	Usual care	Patient navigation	33.6 vs 20.0% overall 26.4 vs 13.0% colonoscopy 7.2 vs 6.5% FOBT	<0.001 overall <0.001 colonoscopy 0.76		
Levy, 2013^40^	[1] Usual care [2] physician chart reminder [3] mailed education + FIT	[4]: [3]+patient navigation	57.2% [4] vs 56.5 [3] overall 19.3% [4] vs 22.0% [3] colonoscopy	<0.0001 overall 0.073 colonoscopy		
Luckmann, 2013^24^	‐	Patient navigation	53.2% overall 68.1% colonoscopy			
McGregor, 2019^62^	Usual care	Patient navigation	79.1 vs 79.8%			
Myers, 2008^55^	‐	Tailored navigation	41%			
Myers, 2013^58^ Daskalakis, 2014^59^ Lairson 2014^60^	[1] Usual care [2] Standard intervention (mailed materials and FOBT)	[3] Tailored navigation	38% [3] vs 12% [1] vs 33% [2] at 6 months 43% [3] vs 18% [1] vs 36% [2] at 12 months	0.001 for both comparisons with [1]	At 6 months: 4.60 (3.02–7.02) vs [1] adjusted 1.25 (0.89–1.75) vs [2] adjusted At 12 months: 3.48 (2.39–5.07) vs [1] 1.30 (0.94–1.81) vs [2] Navigation compared to no navigation: 2.09 (1.26–3.49)	0.001 0.201 0.001 0.118 0.005
Myers, 2014^25^	Standard intervention (mailed materials and FOBT)	Tailored navigation	At 6 months: 38.0% vs 23.7% overall 21.5 vs 15.3% FOBT 16.5 vs 8.4% colonoscopy At 12 months: 43.5% vs 32.2% overall 23.0 vs 18.5% FOBT 20.4 vs 13.7% colonoscopy		2.1 (1.5–2.9) 1.7 (1.2–2.3)	0.001 0.001
Percac‐Lima, 2009^39^ Percac‐Lima, 2014^26^	Usual care	Patient navigation	At 9 months: 27.4 vs 11.9% overall 20.8 vs 9.6% colonoscopy At 5 years: 20.0 vs 11.1% overall 26.0% vs 15.2% among Latinos 26.3% vs 13.9% among non‐English speakers	0.001 0.001 0.001		
Ruggeri, 2020^37^	Baseline	Care gap analysis	47.9% vs 23.2% highest increase in a clinic 43.3% vs 27.68% (average)			
Temucin, 2020^61^	Usual care	Patient navigation	At 3 months: 81.8% vs 9.1% FOBT 14.5% vs 3.6% colonoscopy At 6 months: 83.6% vs 10.9% FOBT 21.8% vs 3.6% colonoscopy	0.000 0.047 0.000 0.004		
Walsh, 2010^56^	[1] Usual care [2] Mailed FOBT and information	[3]: [2] + tailored telephone counselling	25.1% vs 15.1% [2] FOBT 21.4% vs 11.9% [2] any screening test	0.001 0.001		
*Breast cancer*						
Burhansstipanov, 2010^21^	4 newsletters	Patient navigation	54.87% vs 1.5%	<0.05		
Davis, 2014^45^ Davis, 2015^46^	[1] enhanced usual care [2] educational strategy	[3]: [2]+nurse support	At 6 months: 65.8% [3] vs 51.8% [2] With limited literacy: 57.7% [3] vs 55.2% [2] With adequate literacy: 74.3% [3] vs 50.4% [2] At 24–30 months: 48.0% [3] vs 7.1% [2]	0.037 0.17 0.039	At 6 months, [2] reference: 1.37 (1.08–1.74) At 24–30 months, [2] reference: 6.06 (3.63–10.12)	0.01 <0.001
Han, 2009^20^	Baseline	Patient navigation	83.5% vs 51.6% mammography 69.2% vs 46.2% CBE	<0.001 <0.001		
Highfield, 2015^29^	Standard appointment reminder	Tailored counselling reminder			Basic analysis: Unadjusted: 3.38 (1.59–7.21) Adjusted: 3.88 (1.70–8.86) Intention to treat analysis: Unadjusted: 1.84 (1.01–3.35) Adjusted: 2.31 (1.09–4.93)	<0.001 <0.001 <0.05 <0.05
Margulies, 2019^15^	Informational pamphlets	Volunteer run patient navigation	76% vs 42%	<0.05		
Marshall, 2016^31^	Printed education	Printed education + patient navigation	93.3% vs 87.5% 73.4% vs 45.6% among women not screening‐adherent at baseline	<0.001 <0.001	2.26 (1.59–3.42) Among women not screening‐adherent at baseline: 3.63 (2.10–6.26)	<0.001 significant
Molina, 2018^36^	Standard care	Patient navigation	51.4% vs 46.2%	0.04	Adjusted: 1.25 (1.02–1.54)	0.03
Phillips, 2011^22^	Control	Quality improvement patient navigation	87% vs 76% overall 85% vs 70% White 87% vs 78% African American 85% vs 83% Hispanic 88% vs 78% other		Adjusted: 2.5 (1.9–3.2) overall Unadjusted: 2.4 (1.5–4.0) White 1.9 (1.4–2.6) African American 1.2 (0.8–1.8) Hispanic 2.1 (1.3–3.3) other	
Taplin, 2000^51^	[1] Postcard reminder [2] Reminder call	[3]: Motivational call	49.8% vs 35.4% [1] vs 51.8% [2]			
*Cervical cancer*						
Corkrey, 2005^52^	No intervention	Interactive Voice Response (IVR) navigation				
Hewett, 2016^63^	[1] Standard model of service provision	[2] enhanced counselling [3]: [2] + escort	21.3% [2] vs 24.6% [3] vs 4.2% [1]	<0.001 ([1] vs [1, 2] vs [3])	[2] vs [1]: 2.76 (1.94–3.91) [3] vs [1]: 2.98 (2.10–4.22)	<0.001 <0.001
Kitchener, 2016^53^ Kitchener, 2018^54^	[1] Control [2] HPV self‐sampling test sent [3] HPV self‐sampling test offered [4] Timed appointment [5] Choice of nurse navigation or HPV self‐sampling test	[6] Nurse navigation	At 12 months: 14.5% [6] vs 16.2% [1] vs 21.3% [2] vs 16.2% [3] vs 19.8% [4] vs 18.8% [5] At 18 months: 22.8% [6] vs 27.1% [1] vs 30.0% [2] vs 25.8% [3] vs 29.0% [4] vs 30.2% [5]		[1] reference At 12 months: 0.887 (0.670–1.174) [6] 1.091 (0.864–1.378) [5] 1.408 (1.141–1.738) [4] 1.074 (0.871–1.325) [3] 1.512 (1.197–1.910) [2] At 18 months: 0.799 (0.642–0.994) [6] 1.058 (0.869–1.289) [5] 1.191 (0.975–1.456) [4] 1.056 (0.884–1.262) [3] 1.286 (1.056–1.567) [2]	0.401 0.466 0.001 0.505 0.001 0.044 0.573 0.087 0.548 0.012
Paskett, 2011^8^	Usual care	Patient navigation	51.1% vs 42.0% (medical records) 71.3% vs 54.2% (self‐report)	0.135 0.008	Medical records: 1.44 (0.89–2.33) Self‐report: 2.10 (1.22–3.61)	0.135 0.008
Taylor, 2002^18^	[1] Control [2] Direct mail intervention	[3] Outreach worker intervention	39% [3] vs 15% [1] vs 25% [2]	<0.001 [3] vs [1], 0.03 [2] vs [1], 0.02 [3] vs [2]	3.5 (1.9–6.6) [3] vs [1]	<0.001
*Breast and cervical cancer*						
Falk, 2018^34^	Education	Education + patient navigation			Mammography: 2.64 (1.02–1.91) Cytology: 2.72 (2.00–3.69)	<0.001 <0.001
Lee, 2011^64^	‐	Patient navigation	74.3%			
*Breast, cervical and colorectal cancer*						
Beach, 2007^50^	Usual care	Prevention care manager			Adjusted for patient characteristics and baseline up‐to‐date status: Breast cancer: 1.86 (1.39–2.50) Spanish speaking 1.23 (0.85–1.78) English speaking Cervical cancer: 2.18 (1.52–3.13) Spanish speaking 1.25 (0.81–1.91) English speaking Colorectal cancer: 2.12 (1.54–2.90) Spanish speaking 1.62 (1.08–2.45) English speaking	≤0.001 ≤0.001 ≤0.001
Braun, 2015^27^	Control	Patient navigation	61.7% vs 42.4% mammography 57.0% vs 36.4% cytology 43.0% vs 27.2% FS/colonoscopy 20.7% vs 12.6% FOBT	0.003 0.001 <0.001 0.02		
Dietrich, 2007^38^	Outreach programme	Prevention care management			1.16 (0.86–1.57) breast cancer 1.18 (0.82/1.70) cervical cancer 1.69 (1.03–2.77) colorectal cancer	0.33 0.38 0.04
Percac‐Lima, 2016^32^	Usual care	Patient navigation	10.2% vs 6.8% all cancers combined 14.7% vs 11.0% breast cancer 11.1% vs 5.7% cervical cancer 7.6% vs 4.6% colorectal cancer	< 0.001 0.04 0.002 0.01		

Overall, the comparison of patient navigation with usual care (21 studies) or other interventions (10 studies) favoured navigation. Compared to usual care, screening participation was 19.9%^21^ to 250.6%^29^ higher for colorectal cancer, 0.4%^54^ to 160.0%^58^ higher for cervical cancer, and 33.6%^64^ to 45.5%^62^ for breast cancer. Regarding the comparison to educational interventions, participation with patient navigation was 3.3%^26^ to 36.1%^22^ for colorectal cancer, and 6.6%^50^ to 3558.0%^44^ for breast cancer. However, there were a few exceptions: screening participation was lower in patient navigation groups in comparisons against a health‐literacy informed educational intervention in USA,^15^ and against control and other interventions in UK.^56^, ^57^ Another study aimed to assess the impact of introducing patient navigation on social inequalities within the national organized screening programme in France. In this study navigation was found to be more effective in affluent than in deprived strata, entailing that if it was applied to the whole population, it has the potential to aggravate social inequalities in screening participation.^24^, ^25^

## 4 DISCUSSION

To the best of our knowledge, this is the first study to systematically describe the components of patient navigation programmes in breast, cervical and colorectal cancer screening using a specific framework of this intervention. In this systematic review we have identified studies on patient navigation as a single intervention and have described its impact on screening participation when compared against usual care and educational interventions.

Patient navigation increased participation to screening in breast, cervical and colorectal cancer in comparison with usual care and educational interventions alone, in line with the findings from previous publications,^6^, ^8^, ^65^ suggesting patient navigation can improve the effectiveness and outcomes of screening programmes, and advance in health equity.^65^ While many studies included in this review have overcome several previously described limitations (e.g., lack of control group or of randomization to treatment or comparison groups),^6^ there remains an issue of not having a single definition of patient navigation, which in any case is rarely provided.

Moreover, another finding from this work is that studies describing patient navigation interventions could be better reported. Although the ‘who’ (nurse, social worker, lay person, etc.), ‘what’ (what barriers are addressed), ‘how’ (communication channel used) and ‘where’ (setting) aspects of intervention delivery were often described, the intensity of the intervention (number of interactions between navigator and individual, schedule and length) was rarely reported.^66^ This lack of transparency also applied to the control intervention, frequently usual care. Such incompleteness of data hindered the possibility of conducting a meta‐analysis on the impact of patient navigation on breast, cervical and colorectal cancer screening participation, and its association with key components of patient navigation. Therefore, bearing in mind the great variation in the definition of patient navigation, we recommend a standardized reporting of its components that would allow comparison between studies, external validity, replication in different settings and ultimately a better measure of its impact on cancer screening participation. The better reporting of navigation programmes together with a consistent data collection would facilitate sustainability.^67^

A positive finding was that over 84% of studies reported 9 or more components out of 14, being supervision and addressing of social and emotional barriers the least reported. As in a previous review,^68^ the duration of training on patient navigation was quite diverse. We did not find a specific length reported to be adequate, as opposed to 3 days previously described for lay persons in cervical cancer screening.^69^ Compared to a review conducted in the USA,^68^ studies included in ours reported shorter duration of training (half a day vs 12 h). The maximum length of training could not be compared, as few studies reported if training was delivered over time or massed.^22^, ^44^, ^62^ In any case, the length of training is generally linked to the background of trainees, and their expected roles and responsibilities. Another paper from the USA reported a consensus on the domains and competencies of the patient navigation training.^70^ The topics described in the papers in our review included these competencies, but no study covered all.

The local context will determine the importance of each component to achieve a successful patient navigation programme. To plan patient navigation services, we need to know what the population eligible for the selected cancer screening requires through a needs assessment, as Ruggeri et al. did,^41^ which will enable identifying which services it should include.^65^ It is also possible to put in place a patient navigation programme and from its evaluation identify which are the most frequent barriers. Additionally, the least approached barriers were social and emotional, which are related to a lower screening participation^71^ and could entail a delay in seeking medical help.^72^ Moreover, although considered a requirement,^73^ supervision of navigators was seldom described, being more frequently reported in programmes addressing multiple cancer sites screening, probably because of their complexity.

All studies included in this systematic review except one were conducted in high‐income countries. The implementation of patient navigation programmes has been scarcely reported outside USA,^74^ including LMICs. A recent scoping review on this intervention in LMICs in cancer care focused mainly on tertiary level, with only one study on screening. The main services reported were facilitation of the linkage to follow‐up services, coordination of appointments and education to ensure understanding of symptoms and signs. Interestingly, few studies labelled their intervention as patient navigation. Studies evaluating navigation in cancer care reported mainly implementation science outcomes, such as the acceptability, fidelity and feasibility of the intervention,^75^ rarely described in our included studies. Due to a high variability in health care systems across the world, there are limitations to applying the results from high‐income countries to LMICs. More research is needed in these settings to understand patient navigation in cancer screening with a global perspective.

Previous systematic reviews focused on USA and Canada only.^6^, ^8^ The inclusion of studies conducted in countries other than these two is one of the strengths of our systematic review. Other strengths are the search in three databases, including both medical and social databases, no language restriction and the use of a framework to assess the reporting of elements of patient navigation programmes.

The main limitation of this research is that the systematic search was not initially developed to assess patient navigation, as it included only “patient navigation” and “patient‐centred care” as search terms. However, when assessing the full texts, we were broad in the consideration of the term, as screening practitioners, prevention managers and care gap analysts have been included as navigators. Additionally, the inclusion of studies measuring self‐reported participation to screening may have overestimated the impact of navigation.

In conclusion, patient navigation is effective in increasing participation in breast, cervical and colorectal cancer screening, which can improve the effectiveness and outcomes of screening programmes. A standardized reporting of patient navigation and its components would allow its replication and a better measure of its impact. The local context and needs will determine the importance of each component and will enable the design of a successful patient navigation programme.

## AUTHOR CONTRIBUTIONS

**Isabel Mosquera:** Conceptualization (equal); data curation (lead); formal analysis (equal); investigation (lead); methodology (equal); validation (lead); visualization (lead); writing – original draft (lead). **Adam Todd:** Conceptualization (equal); methodology (equal); supervision (equal); writing – review and editing (equal). **Mirza Balaj:** Conceptualization (equal); methodology (equal); writing – review and editing (equal). **Li Zhang:** Investigation (supporting); writing – review and editing (equal). **Sara Benitez Majano:** Data curation (supporting); investigation (supporting); writing – review and editing (equal). **Keitly Mensah:** Investigation (supporting); writing – review and editing (equal). **Terje Andreas Eikemo:** Funding acquisition (lead); writing – review and editing (equal). **Partha Basu:** Funding acquisition (supporting); writing – review and editing (equal). **Andre L Carvalho:** Conceptualization (equal); formal analysis (equal); investigation (lead); methodology (equal); supervision (equal); validation (supporting); writing – review and editing (equal).

## CONFLICT OF INTEREST

The authors declare no conflicts of interest.

## DISCLAIMER

Where authors are identified as personnel of the International Agency for Research on Cancer/World Health Organization, the authors alone are responsible for the views expressed in this article and they do not necessarily represent the decisions, policy or views of the International Agency for Research on Cancer/World Health Organization.

## Data Availability

The datasets generated and/or analyzed during the current study are included within the article and the supplementary information.

## APPENDIX 1 SEARCH STRATEGY USED IN WEB OF SCIENCE CORE COLLECTION

#1. TS = (breast) OR TS = (mammary)

#2. TS = (precancer* OR cancer* OR neoplas* OR tumor OR tumors OR tumour OR tumours OR carcinoma* OR adenocarcinoma* OR “adeno carcinoma*” OR adenoma* OR malignan* OR lesion*)

#3. #2 AND #1

#4. TS = (“cervical intraepithelial neoplas*” OR “uterine cervical neoplas*” OR “uterine cervical dysplas*” OR “atypical squamous cells of the cervix” OR “squamous intraepithelial lesions of the cervix”)

#5. TS = (“cervix uteri” OR cervical OR cervix OR cervixes OR cervices OR cervico*)

#6. TS = (precancer* OR cancer* OR neoplas* OR dysplas* OR dyskarios* OR tumor OR tumors OR tumour OR tumours OR carcinoma* OR adenocarcinoma* OR “adeno carcinoma*” OR adenoma* OR malignan* OR lesion* OR squamous OR “small cell” OR “large cell”)

#7. #6 AND #5

#8. #7 OR #4

#9. TS = (“colorectal neoplas*” OR “colonic neoplas*” OR “rectal neoplas*”)

#10. TS = (colon OR colonic OR bowel OR rectal OR rectum OR sigmoid OR anal OR anus)

#11. TS = (precancer* OR cancer* OR neoplas* OR dysplas* OR tumor OR tumors OR tumour OR tumours OR carcinoma* OR adenocarcinoma* OR “adeno carcinoma*” OR adenoma* OR malignan* OR lesion*)

#12. #11 AND #10

#13. #12 OR #9

#14. #13 OR #8 OR #3

#15. TS = (“socioeconomic factor*”) OR TS = (“educational status”) OR TS = (“educational level”) OR TS = (employment) OR TS = (occupation*) OR TS = (income) OR TS = (poverty) OR TS = (“social class*”)

#16. TS = (“socioeconomic inequalit*”) OR TS = (“socioeconomic inequit*”) OR TS = (“socioeconomic equalit*”) OR TS = (“socioeconomic equit*”) OR TS = (“health status disparit*”) OR TS = (“health disparit*”) OR TS = (“healthcare disparit*”) OR TS = (“health care disparit*”) OR TS = (“health inequalit*”) OR TS = (“health inequit*”) OR TS = (“health equalit*”) OR TS = (“health equit*”)

#17. TS = (“health literacy”) OR TS = (“depriv*”) OR TS = (gender) OR TS = (“minority group*”) OR TS = (“ethnic group*”) OR TS = (“vulnerable population*”) OR TS = (“disadvantaged population*”) OR TS = (“underserved population*”) OR TS = (“population group*”) OR TS = (“urban population*”) OR TS = (“suburban population*”) OR TS = (“rural population*”)

#18. TS = (awareness) OR TS = (access) OR TS = (barrier*) OR TS = (obstacle*) OR TS = (challenge*) OR TS = (gap) OR TS = (gaps) OR TS = (facilitator*) OR TS = (“patient acceptance of health care”) OR TS = (“patient acceptance of healthcare”) OR TS = (“patient dropout*”) OR TS = (“physician patient relation*”) OR TS = (“health knowledge”) OR TS = (attitude*) OR TS = (practice*) OR TS = (“persuasive communication”) OR TS = (“health behaviour*”) OR TS = (“health behavior*”)

#19. #18 OR #17 OR #16 OR #15

#20. TS = (“mass screening”) OR TS = (“population surveillance”) OR TS = (“screening and testing”) OR TS = (“early diagnosis”) OR TS = (“secondary prevention”) OR TS = (“early detection”)

#21. #20 AND #19 AND #14

#22. TS = (“cost shar*”) OR TS = (“reduced copay*”) OR TS = (“reduced cost*”) OR TS = (schedul*) OR TS = (transport*) OR TS = (“mobile health unit*”) OR TS = (“mobile unit*”) OR TS = (technolog*) OR TS = (“cell phone*”) OR TS = (“mobile phone*”) OR TS = (“text messag*”) OR TS = (“short message service”) OR TS = (sms) OR TS = (“communication barrier*”) OR TS = (“language barrier*”) OR TS = (“patient navigat*”) OR TS = (“patient centered care”) OR TS = (“patient‐centered care”) OR TS = (selfsampling) OR TS = (“self sampling”) OR TS = (choice)

#23. TS = (intervention*) OR TS = (implement*) OR TS = (action*) OR TS = (experiment*) OR TS = (“referral and consultation”)

#24. #23 OR #22

#25. TS = (“patient participation”) OR TS = (“community participation”) OR TS = (“stakeholder participation”) OR TS = (participat*) OR TS = (“patient compliance”) OR TS = (uptake) OR TS = (adherence) OR TS = (coverage)

#26. TS = (“voluntary program*”) OR TS = (voluntary) OR TS = (attendance) OR TS = (utilization) OR TS = (utilisation) OR TS = (“health promot*”) OR TS = (impact*) OR TS = (effect*) OR TS = (performance) OR TS = (“health care outcome assess*”) OR TS = (“healthcare outcome assess*”) OR TS = (examinat*) OR TS = (monitor*) OR TS = (“program* evaluat*”)

#27. #26 OR #25

#28. #27 AND #24 AND #21

#29. #27 AND #24 AND #21

Refined by: PY = (2000–2020)

## APPENDIX 2 TABLE OF INCLUDED STUDIES BY CANCER SITE

The studies are provided in alphabetical order. However, when more than one paper describes a study, they have been placed consecutively.

**Table:**

First author, year	Period of analyzed data	Region, country	Population/sample % women if applicable age	Measure of socioeconomic position in inclusion criteria	Measure of socioeconomic position in analysis	Screening method evaluated	Study design (number of arms)	Quality assessment
*Colorectal cancer*								
Arnold, 2016^41^	May 2008–August 2011	Louisiana, USA	206 participants with negative FOBT in first and second round 80% women 50–85 years	Income, insurance	Health literacy	FOBT	Cluster RCT (3 arms)	Poor
Braun, 2005^19^	2002–2003	Hawaii, USA	121 Native Hawaiians 70% women in intervention and 75% in control ≥50 years	Ethnicity/race	–	FOBT	RCT (2 arms)	Poor
Cole, 2017^33^	2010–2013	New York city, USA	731 self‐identified Black men ≥50 years	Ethnicity/race	–	Self‐reported FOBT or colonoscopy	RCT (3 arms)	Good
Davis, 2013^43^ Davis, 2014^44^	May 2008 – August 2011	Louisiana, USA	961 patients 77% women 50–85 years 461 patients with negative initial FOBT 75% women 50–85 years	Geographic area (rural)	Literacy	gFOBT	Quasi‐experimental (3 arms)	Fair
DeGroff, 2017^42^	September 2012–December 2014	Massachusetts, USA	843 adults (429 in intervention and 427 in control) 57.1% women 50–75 years	Income, ethnicity/race	–	Colonoscopy	RCT (2 arms)	Fair
Dietrich, 2013^49^	December 2008 – July 2010	New York, USA	2240 women (562 in intervention and 1678 in control) 50–63 years	Primary language	–	FOBT, colonoscopy, sigmoidoscopy, or barium enema	RCT (2 arms)	Fair
Enard, 2015^28^	March 2007–December 2010	USA	2084 Latino Medicare fee‐for service (FFS) enrollees (1044 in intervention and 1040 in comparison) ≥40 years 54.8% women in final analysis	Ethnicity/race	–	Self‐reported FOBT, colonoscopy or sigmoidoscopy	RCT (2 arms)	Poor
Green, 2013^57^	August 2008 – November 2009	Washington, USA	4675 adults (653 in arm 1, 629 in arm 2, 615 in arm 3 and 647 in arm 4) 50–73 years	–	–	FOBT, colonoscopy, sigmoidoscopy	Cluster RCT	Good
Guillaume, 2017^16^ De Mil, 2018^17^	April 2011–April 2013	3 departments in France	14,373 subjects in the intervention group and 14,556 control subjects 50–74 years	Material deprivation, rural/urban	Material deprivation, rural/urban	FOBT	Cluster RCT (2 arms)	Poor
Horne, 2015^30^	April 2006 – June 2010	Baltimore city, USA	1220 African Americans 72.8% women in intervention, 72.3% in control 65–75 years	Ethnicity/race	Health literacy	Colonoscopy/sigmoidoscopy, FOBT	RCT (2 arms)	Poor
Jandorf, 2013^23^	May 2008 – September 2011	USA	532 African Americans > 50 years	Ethnicity/race	–	Colonoscopy	RCT (3 arms)	Poor
Kim, 2018^35^	August 2016–April 2017	Chicago, USA	536 individuals in intervention and 2713 in control Approx. 60% women in both arms 50–75 years	Ethnicity/race	–	Colonoscopy	Cost‐effectiveness study, controlled trial (2 arms)	Poor
Lasser, 2009^48^	October 2007	Massachusetts, USA	55 patients 63.4% women in intervention, 75.6% in control 52–80 years	Primary language	–	FOBT, colonoscopy, sigmoidoscopy or barium enema	RCT (2 arms)	Poor
Lasser, 2011^47^	September 2008 – March 2010	Massachusetts, USA	465 primary care patients (235 in intervention −60.4% women, 230 in control −62.6% women) 52–74 years	Primary language	Age, ethnicity/race, primary language, health insurance coverage	FOBT, colonoscopy, sigmoidoscopy or barium enema	RCT (2 arms)	Good
Levy, 2013^40^	December 2008–April 2011	Iowa, USA	743 primary care patients (185 in usual care, 185 in physician chart reminder, 186 in mailed education/FIT, 187 in patient navigation) aged 50.8–53.5% women in each arm 52 to 79 years	Geographic area (rural), income	–	FIT, colonoscopy	RCT (4 arms)	Fair
Luckmann, 2013^24^	February 2006–May 2007	Massachusetts, USA	362 primary care patients 63% women 50–79 years	Ethnicity/race, socioeconomic status, geographic area (urban, rural)	–	FOBT, colonoscopy, sigmoidoscopy, barium enema or virtual colonoscopy	After only (1 arm)	Poor
McGregor, 2019^62^	Recruitment: May–October 2015	Tyne and Wear, England, UK	152 not confirming or attending appointment (109 in intervention −53.2%‐, 43 in control −53.5%)	–	–	Flexible sigmoidoscopy	RCT (2 arms)	Poor
Myers, 2008^55^	–	Delaware, USA	154 primary care practice patients 57% women ≥50 years	–	–	FOBT, colonoscopy, sigmoidoscopy or barium enema	After only study (1 arm)	Fair
Myers, 2013^58^ Daskalakis, 2014^59^ Lairson, 2014^60^	2007–2011	Delaware, USA	945 primary care patients 66% women in tailored navigation intervention (*n* = 312), 64% in standard intervention (*n* = 316) and 57% in control (*n* = 317) 50–79 years	–	–	Self‐reported FOBT, colonoscopy, sigmoidoscopy or barium enema	RCT (3 arms)	Fair
Myers, 2014^25^	December 2008–October 2011 (identification of patients)	Philadelphia, USA	764 African Americans (384 in intervention and 380 in control) 72.7% women in intervention and 64.1 in control 50–75 years	Ethnicity/race	–	FOBT, colonoscopy, sigmoidoscopy or barium enema	RCT (2 arms)	Fair
Percac‐Lima, 2009^39^ Percac‐Lima, 2014^26^	January–October 2007 2006–2010	USA	1223 patients (409 in intervention and 814 in control) 60% women 52–79 years 3115 patients in one practice and 43,905 patients in the other practices of the same network	Income, primary language Income, ethnicity/race	– Primary language	FOBT, colonoscopy, sigmoidoscopy or barium enema	RCT (2 arms) Controlled trial (2 arms)	Fair
Ruggeri, 2020^37^	2016–2018	USA	452 clinic patients 50–75 years	Ethnicity/race, insurance	–	FIT	Before‐after	Poor
Temucin, 2020^61^	–	Istanbul, Turkey	110 individuals (55 in intervention and 55 in control) registered at family health centres 50–70 years 65.5% women	–	–	FOBT, colonoscopy	RCT (2 arms)	Good
Walsh, 2010^56^	Baseline survey: 2005–2006, follow‐up survey: 2006–2007	California, USA	1358 individuals 69.7% women in patient navigation, 69.0% in intervention and 69.1% in control 50–79 years	Ethnicity/race	Ethnicity/race	Self‐reported FOBT, colonoscopy, sigmoidoscopy	RCT (3 arms)	Poor
*Breast cancer*								
Burhansstipanov, 2010^21^	(5 years: training of navigators started in 2001; study ongoing in 2005)	Denver, USA	313 women (113 in intervention and 200 in control) 40–85 years	Ethnicity/race, income	–	Mammography	RCT (2 arms)	Poor
Davis, 2014^45^ Davis, 2015^46^	May 2008 – August 2011	Louisiana, USA	1181 patients (323 in enhanced care, 355 in education, 503 in nurse support) ≥40 years 624 patients with negative initial mammography (172 in enhanced care, 154 in education, 298 in nurse support) ≥40 years	Geographic area (rural)	Literacy	Mammography	Cluster RCT (3 arms)	Poor
Han, 2009^20^	–	USA	100 Korean American women 40–80 years	Ethnicity/race	–	Mammography, CBE	After only (1 arm)	Poor
Highfield, 2015^29^	February – December 2012	Houston area, USA	151 African American women 36–64 years	Ethnicity/race, income, insurance	–	Mammography	Initially RCT and after quasi experimental Type I hybrid design (2 arms)	Poor
Margulies, 2019^15^	3 weeks after September 2015	USA	49 women (25 in intervention and 24 in control) 40–76 years	Income	Employment	Mammography	RCT (2 arms)	Fair
Marshall, 2016^31^	April 2006–December 2010	Baltimore, USA	1905 African American Medicare beneficiaries ≥ 65 years	Ethnicity/race	Health literacy	Mammography	RCT	Poor
Molina, 2018^36^	2011–2014 initial study	Chicago, USA	2536 women with access to primary care (741 in intervention and 1795 in control) 50–74 years	Income, ethnicity/race	–	Mammography	RCT (2 arms)	Fair
Phillips, 2011^22^	February–November 2008	USA	3895 women in hospital‐based primary care practice (1817 in intervention and 2078 in control) 51–70 years	Ethnicity/race, income	Ethnicity/race, insurance, education	Mammography	Cluster RCT (2 arms)	Poor
Taplin, 2000^51^	–	Seattle, USA	1765 women 50–79 years	–	–	Mammography	Randomized trial	Fair
*Cervical cancer*								
Corkrey, 2005^52^	April–July 2001	New South Wales, Australia	18–69 years	–	–	Cytology	RCT (2 arms)	Poor
Hewett, 2016^63^	2013	Lusaka and Chipata districts, Zambia	2043 women (678 in arm 1, 685 in arm 2, 680 in arm 3) ≥18 years	–	–	–	RCT (3 arms)	Fair
Kitchener, 2016^53^ Kitchener, 2018^54^	April 2013 – November 2014 (phase II)	Greater Manchester and Grampian, UK	10,126 women non‐attenders in phase I of study (1007 in nurse navigation, 1277 in choice between nurse navigation and HPV self‐sampling) 25 years	–	–	HPV self‐sampling, cytology	Cluster RCT (6 arms)	Fair
Paskett, 2011^8^	May 2005 – February 2009	Ohio, USA	280 women (143 in intervention and 137 in control) ≥ 18 years	Geographic area	–	Cytology	RCT (2 arms)	Poor
Taylor, 2002^18^	1999	Seattle, USA, and Vancouver, Canada	482 Chinese women 20–69 years	Ethnicity/race	–	Cytology	RCT	Poor
*Breast and cervical cancer*								
Falk, 2018^34^	March 2012 – February 2015	Rural and Border Texas, USA	2689 women self‐identified as African American, Latina or non‐Hispanic white 18–99 years	Geographic area, income, insurance	Ethnicity/race, primary language	Mammography, cytology	Controlled trial (2 arms)	Poor
Lee, 2011^64^	September 2008 – October 2009	Chuncheon city, South Korea	210 women >40 years	–	–	–	Before‐after (1 arm)	Fair
*Breast, cervical and colorectal cancer*								
Beach, 2007^50^	November 2001–April 2004	USA	1346 women 50–69 years	Language preference	Language preference	Mammography, cytology, and flexible sigmoidoscopy/colonoscopy	RCT (2 arms)	Fair
Braun, 2015^27^	2006–2009	Hawaii, USA	488 Asian and Pacific Islander Medicare beneficiaries (242 in intervention and 246 in control) Approx. 53% female	Ethnicity/race	–	Mammography, cytology, and flexible sigmoidoscopy/colonoscopy	RCT (2 arms)	Poor
Dietrich, 2007^38^	May – December 2005	New York city, USA	1316 women (663 in intervention and 653 in control) not up to date with at least on cancer screening 40–69 years	Income	–	mammography, cytology, FOBT, sigmoidoscopy, double contrast barium enema, colonoscopy	Cluster RCT (2 arms)	Poor
Percac‐Lima, 2016^32^	April – December 2014	USA	1612 individuals overdue for at least one screening (792 in intervention and 820 in control) 60.5% women in intervention and in control 21–64 years (breast and cervical cancer) 50–75 years (colorectal cancer)	–	Ethnicity/race, primary language, insurance	Mammogram, breast magnetic resonance imaging, cytology, HPV testing, colonoscopy, sigmoidoscopy, barium enema, colonography	RCT (2 arms)	Fair

Abbreviations: CBE, clinical breast examination; FIT, fecal immunochemical test; FOBT, fecal occult blood test; gFOBT, guaiac FOBT; RCT, randomized controlled trial.

## APPENDIX 3 COMPONENTS OF PATIENT NAVIGATION PROGRAMMES DESCRIBED IN THE INCLUDED STUDIES, OVERALL AND BY CANCER SITE

**Table:**

Components of patient navigation programme	Overall *N* (%)	Colorectal cancer *N* (%)	Breast cancer *N* (%)	Cervical cancer *N* (%)	Multiple cancer sites *N* (%)
Programme goals	44 (100)	24 (100)	9 (100)	5 (100)	6 (100)
Community characteristics	44 (100)	24 (100)	9 (100)	5 (100)	6 (100)
Point of intervention	44 (100)	24 (100)	9 (100)	5 (100)	6 (100)
Setting	43 (97.7)	24 (100)	9 (100)	4 (80)	6 (100)
Monitoring and evaluation (other than screening participation)	43 (97.7)	23 (95.8)	9 (100)	5 (100)	6 (100)
Communication	42 (95.5)	23 (95.8)	9 (100)	5 (100)	5 (83.3)
Background and qualifications	35 (81.4)^a^	18 (75)	8 (88.9)	3 (75)^a^	6 (100)
Training	34 (79.1)^a^	18 (75)	9 (100)	3 (75)^a^	4 (66.7)
Address educational barriers	28 (63.6)	16 (66.7)	6 (66.7)	2 (40)	4 (66.7)
Address health system barriers	27 (61.4)	15 (62.5)	6 (66.7)	2 (40)	4 (66.7)
Theoretical framework	21 (47.7)	12 (50)	5 (55.6)	1 (20)	3 (50.0)
Address individual barriers	21 (47.7)	10 (41.7)	6 (66.7)	3 (60)	2 (33.3)
Supervision	16 (36.4)	9 (37.5)	3 (33.3)	1 (20)	3 (50.0)
Address social and emotional barriers	11 (25.0)	5 (20.8)	2 (22.2)	2 (40)	2 (33.3)

^a^ Excludes one study where this component was not applicable.

## APPENDIX 4 COMPONENTS OF PATIENT NAVIGATION PROGRAMMES IN THE INCLUDED STUDIES BY CANCER SITE

**Table:**

First author, year	Theor. framework	Progr. goals	Community character.	Point of interv.	Setting	Address health system barriers	Address individual barriers	Address educational barriers	Address social and emotional barriers	Commun. method	Navigator background and qualification	Training	Supervision	Monitoring and evaluation (other than screening participation)
*Colorectal cancer*														
Arnold, 2016^41^	✓	✓	✓	✓	✓	✓				✓	✓			✓
Braun, 2005^19^	✓	✓	✓	✓	✓	✓	✓		✓	✓				✓
Cole, 2017^33^		✓	✓	✓	✓		✓	✓	✓	✓				✓
Davis, 2013^43^ Davis, 2014^44^	✓	✓	✓	✓	✓	✓	✓	✓		✓	✓	✓		✓
DeGroff, 2017^42^	✓	✓	✓	✓	✓	✓	✓	✓	✓	✓	✓	✓	✓	✓
Dietrich, 2013^49^		✓	✓	✓	✓	✓	✓			✓	✓	✓	✓	✓
Enard, 2015^28^		✓	✓	✓	✓		✓	✓		✓	✓	✓		✓
Green, 2013^57^		✓	✓	✓	✓	✓				✓	✓			✓
Guillaume, 2017^16^ De Mil, 2018^17^		✓	✓	✓	✓			✓		✓	✓	✓	✓	✓
Horne, 2015^30^		✓	✓	✓	✓							✓		✓
Jandorf, 2013^23^	✓	✓	✓	✓	✓	✓	✓	✓	✓	✓	✓	✓		✓
Kim, 2018^35^		✓	✓	✓	✓			✓		✓	✓	✓	✓	✓
Lasser, 2009^48^	✓	✓	✓	✓	✓			✓		✓	✓	✓	✓	✓
Lasser, 2011^47^	✓	✓	✓	✓	✓	✓	✓	✓		✓	✓	✓	✓	✓
Levy, 2013^40^		✓	✓	✓	✓			✓		✓				✓
Luckmann, 2013^24^	✓	✓	✓	✓	✓	✓	✓	✓		✓	✓	✓		✓
McGregor, 2019^62^		✓	✓	✓	✓	✓			✓	✓	✓	✓	✓	✓
Myers, 2008^55^	✓	✓	✓	✓	✓	✓		✓		✓		✓		✓
Myers, 2013^58^ Daskalakis, 2014^59^ Lairson, 2014^60^	✓	✓	✓	✓	✓	✓		✓		✓	✓	✓		✓
Myers, 2014^25^	✓	✓	✓	✓	✓			✓		✓		✓		✓
Percac‐Lima, 2009^39^ Percac‐Lima, 2014^26^		✓	✓	✓	✓	✓	✓	✓		✓	✓	✓	✓	✓
Ruggeri, 2020^37^		✓	✓	✓	✓	✓				✓	✓			
Temucin, 2020^61^	✓	✓	✓	✓	✓	✓		✓		✓	✓	✓		✓
Walsh, 2010^56^		✓	✓	✓	✓					✓	✓	✓	✓	✓
*Breast cancer*														
Burhansstipanov, 2010^21^	✓	✓	✓	✓	✓		✓	✓		✓	✓	✓		✓
Davis, 2014^45^ Davis, 2015^46^	✓	✓	✓	✓	✓	✓		✓		✓	✓	✓		✓
Han, 2009^20^	✓	✓	✓	✓	✓	✓	✓	✓		✓	✓	✓		✓
Highfield, 2015^29^	✓	✓	✓	✓	✓					✓		✓	✓	✓
Margulies, 2019^15^		✓	✓	✓	✓		✓	✓		✓	✓	✓		✓
Marshall, 2016^31^		✓	✓	✓	✓	✓		✓		✓	✓	✓	✓	✓
Molina, 2018^36^		✓	✓	✓	✓	✓	✓		✓	✓	✓	✓		✓
Phillips, 2011^22^		✓	✓	✓	✓	✓	✓			✓	✓	✓		✓
Taplin, 2000^51^	✓	✓	✓	✓	✓	✓	✓	✓	✓	✓	✓	✓	✓	✓
*Cervical cancer*														
Corkrey, 2005^52^		✓	✓	✓			✓	✓	✓	✓	NA	NA		✓
Hewett, 2016^63^		✓	✓	✓	✓		✓			✓				✓
Kitchener, 2016^53^ Kitchener, 2018^54^		✓	✓	✓	✓	✓				✓	✓	✓		✓
Paskett, 2011^8^	✓	✓	✓	✓	✓			✓		✓	✓	✓	✓	✓
Taylor, 2002^18^		✓	✓	✓	✓	✓	✓		✓	✓	✓	✓		✓
*Breast and cervical cancer*														
Falk, 2018^34^	✓	✓	✓	✓	✓					✓	✓			✓
Lee, 2011^64^	✓	✓	✓	✓	✓			✓	✓		✓	✓	✓	✓
*Breast, cervical and colorectal cancer*														
Beach, 2007^50^		✓	✓	✓	✓	✓			✓	✓	✓			✓
Braun, 2015^27^	✓	✓	✓	✓	✓	✓	✓	✓		✓	✓	✓	✓	✓
Dietrich, 2007^38^		✓	✓	✓	✓	✓		✓		✓	✓	✓		✓
Percac‐Lima, 2016^32^		✓	✓	✓	✓	✓	✓	✓		✓	✓	✓	✓	✓

Abbreviations: Character., characteristics; commun., communication; interv., intervention; NA, not applicable; progr., programme, theor., theoretical.

## References

[1] Lortet‐Tieulent J , Georges D , Bray F , Vaccarella S . Profiling global cancer incidence and mortality by socioeconomic development. Int J Cancer. 2020;147:3029‐3036. 32449164 10.1002/ijc.33114

[2] Gini A , Jansen EEL , Zielonke N , et al. Impact of colorectal cancer screening on cancer‐specific mortality in Europe: a systematic review. Eur J Cancer. 2020;127:224‐235. 31932176 10.1016/j.ejca.2019.12.014

[3] Jansen EEL , Zielonke N , Gini A , et al. Effect of organised cervical cancer screening on cervical cancer mortality in Europe: a systematic review. Eur J Cancer. 2020;127:207‐223. 31980322 10.1016/j.ejca.2019.12.013

[4] Zielonke N , Gini A , Jansen EEL , et al. Evidence for reducing cancer‐specific mortality due to screening for breast cancer in Europe: a systematic review. Eur J Cancer. 2020;127:191‐206. 31932175 10.1016/j.ejca.2019.12.010

[5] Freeman HP . The history, principles, and future of patient navigation: commentary. Semin Oncol Nurs. 2013;29:72‐75. 23651676 10.1016/j.soncn.2013.02.002

[6] Wells KJ , Battaglia TA , Dudley DJ , et al. Patient navigation: state of the art or is it science? Cancer. 2008;113:1999‐2010. 18780320 10.1002/cncr.23815 PMC2679696

[7] DeGroff A , Coa K , Morrissey KG , Rohan E , Slotman B . Key considerations in designing a patient navigation program for colorectal cancer screening. Health Promot Pract. 2014;15:483‐495. 24357862 10.1177/1524839913513587 PMC4618321

[8] Paskett ED , McLaughlin JM , Lehman AM , Katz ML , Tatum CM , Oliveri JM . Evaluating the efficacy of lay health advisors for increasing risk‐appropriate pap test screening: a randomized controlled trial among Ohio Appalachian women. Cancer Epidemiol Biomarkers Prev. 2011;20:835‐843. 21430302 10.1158/1055-9965.EPI-10-0880 PMC3089673

[9] Bernardo BM , Zhang X , Beverly Hery CM , Meadows RJ , Paskett ED . The efficacy and cost‐effectiveness of patient navigation programs across the cancer continuum: a systematic review. Cancer. 2019;125:2747‐2761. 31034604 10.1002/cncr.32147

[10] IARC . Breast Cancer Screening. IARC Handbooks of Cancer Prevention Volume 15. IARC; 2014.

[11] IARC . Colorectal Cancer Screening. IARC Handbooks of Cancer Prevention Volume 17. IARC; 2019.

[12] IARC . Cervical Cancer Screening. IARC Handbooks of Cancer Prevention Volume 18. IARC; 2022.

[13] National Heart, Lung, and Blood Institute . Study quality assessment tools; 2013. Accessed January 2022. https://www.nhlbi.nih.gov/health‐topics/study‐quality‐assessment‐tools

[14] Popay J , Roberts H , Sowden A , et al. Guidance of the conduct of narrative synthesis in systematic reviews. A product from the ESRC Methods programme. 2006.

[15] Arnold CL , Rademaker A , Wolf MS , Liu D , Hancock J , Davis TC . Third annual fecal occult blood testing in community health clinics. Am J Health Behav. 2016;40:302‐309. 27103409 10.5993/AJHB.40.3.2 PMC4955393

[16] Braun KL , Fong M , Kaanoi ME , Kamaka ML , Gotay CC . Testing a culturally appropriate, theory‐based intervention to improve colorectal cancer screening among native Hawaiians. Prev Med. 2005;40:619‐627. 15850857 10.1016/j.ypmed.2004.09.005 PMC2914227

[17] Cole H , Thompson HS , White M , et al. Community‐based, preclinical patient navigation for colorectal cancer screening among older black men recruited from barbershops: the MISTER B trial. Am J Public Health. 2017;107:1433‐1440. 28727540 10.2105/AJPH.2017.303885 PMC5551599

[18] Davis T , Arnold C , Rademaker A , et al. Improving colon cancer screening in community clinics. Cancer. 2013;119:3879‐3886. 24037721 10.1002/cncr.28272 PMC3805687

[19] Davis TC , Arnold CL , Bennett CL , et al. Strategies to improve repeat fecal occult blood testing cancer screening. Cancer Epidemiol Biomarkers Prev. 2014;23:134‐143. 24192009 10.1158/1055-9965.EPI-13-0795 PMC3894742

[20] DeGroff A , Schroy PC III , Morrissey KG , et al. Patient navigation for colonoscopy completion: results of an RCT. Am J Prev Med. 2017;53:363‐372. 28676254 10.1016/j.amepre.2017.05.010 PMC8855664

[21] Dietrich AJ , Tobin JN , Robinson CM , et al. Telephone outreach to increase colon cancer screening in Medicaid managed care organizations: a randomized controlled trial. Ann Fam Med. 2013;11:335‐343. 23835819 10.1370/afm.1469 PMC3704493

[22] Enard KR , Nevarez L , Hernandez M , et al. Patient navigation to increase colorectal cancer screening among Latino Medicare enrollees: a randomized controlled trial. Cancer Causes Control. 2015;26:1351‐1359. 26109462 10.1007/s10552-015-0620-6 PMC5215648

[23] Green BB , Wang CY , Anderson ML , et al. An automated intervention with stepped increases in support to increase uptake of colorectal cancer screening: a randomized trial. Ann Intern Med. 2013;158:301‐311. 23460053 10.7326/0003-4819-158-5-201303050-00002 PMC3953144

[24] Guillaume E , Dejardin O , Bouvier V , et al. Patient navigation to reduce social inequalities in colorectal cancer screening participation: a cluster randomized controlled trial. Prev Med. 2017;103:76‐83. 28823681 10.1016/j.ypmed.2017.08.012

[25] De Mil R , Guillaume E , Guittet L , et al. Cost‐effectiveness analysis of a navigation program for colorectal cancer screening to reduce social health inequalities: a French cluster randomized controlled trial. Value Health. 2018;21:685‐691. 29909873 10.1016/j.jval.2017.09.020

[26] Horne HN , Phelan‐Emrick DF , Pollack CE , et al. Effect of patient navigation on colorectal cancer screening in a community‐based randomized controlled trial of urban African American adults. Cancer Causes Control. 2015;26:239‐246. 25516073 10.1007/s10552-014-0505-0 PMC4370183

[27] Jandorf L , Braschi C , Ernstoff E , et al. Culturally targeted patient navigation for increasing African Americans' adherence to screening colonoscopy: a randomized clinical trial. Cancer Epidemiol Biomarkers Prev. 2013;22:1577‐1587. 23753039 10.1158/1055-9965.EPI-12-1275 PMC3769457

[28] Kim KE , Randal F , Johnson M , et al. Economic assessment of patient navigation to colonoscopy‐based colorectal cancer screening in the real‐world setting at the University of Chicago Medical Center. Cancer. 2018;124:4137‐4144. 30359474 10.1002/cncr.31690 PMC6263829

[29] Lasser KE , Murillo J , Medlin E , et al. A multilevel intervention to promote colorectal cancer screening among community health center patients: results of a pilot study. BMC Fam Pract. 2009;10:37. 19480698 10.1186/1471-2296-10-37 PMC2694166

[30] Lasser KE , Murillo J , Lisboa S , et al. Colorectal cancer screening among ethnically diverse, low‐income patients: a randomized controlled trial. Arch Intern Med. 2011;171:906‐912. 21606094 10.1001/archinternmed.2011.201

[31] Levy BT , Xu Y , Daly JM , Ely JW . A randomized controlled trial to improve colon cancer screening in rural family medicine: an Iowa Research Network (IRENE) study. J Am Board Fam Med. 2013;26:486‐497. 24004700 10.3122/jabfm.2013.05.130041

[32] Luckmann R , Costanza ME , Rosal M , White MJ , Cranos C . Referring patients for telephone counseling to promote colorectal cancer screening. Am J Manag Care. 2013;19:702‐708. 24304253

[33] McGregor LM , Skrobanski H , Ritchie M , et al. Using specialist screening practitioners (SSPs) to increase uptake of bowel scope (flexible sigmoidoscopy) screening: results of a feasibility single‐stage phase II randomised trial. BMJ Open. 2019;9:e023801. 10.1136/bmjopen-2018-023801 PMC6398706 30772850

[34] Myers RE , Hyslop T , Sifri R , et al. Tailored navigation in colorectal cancer screening. Med Care. 2008;46:S123‐S131. 18725824 10.1097/MLR.0b013e31817fdf46

[35] Myers RE , Bittner‐Fagan H , Daskalakis C , et al. A randomized controlled trial of a tailored navigation and a standard intervention in colorectal cancer screening. Cancer Epidemiol Biomarkers Prev. 2013;22:109‐117. 23118143 10.1158/1055-9965.EPI-12-0701 PMC5537598

[36] Daskalakis C , Vernon SW , Sifri R , et al. The effects of test preference, test access, and navigation on colorectal cancer screening. Cancer Epidemiol Biomarkers Prev. 2014;23:1521‐1528. 24813819 10.1158/1055-9965.EPI-13-1176 PMC4119540

[37] Lairson DR , DiCarlo M , Deshmuk AA , et al. Cost‐effectiveness of a standard intervention versus a navigated intervention on colorectal cancer screening use in primary care. Cancer. 2014;120:1042‐1049. 24435411 10.1002/cncr.28535 PMC3961516

[38] Myers RE , Sifri R , Daskalakis C , et al. Increasing colon cancer screening in primary care among African Americans. J Natl Cancer Inst. 2014;106:dju344. 25481829 10.1093/jnci/dju344 PMC4817126

[39] Percac‐Lima S , Grant RW , Green AR , et al. A culturally tailored navigator program for colorectal cancer screening in a community health center: a randomized, controlled trial. J Gen Intern Med. 2009;24:211‐217. 19067085 10.1007/s11606-008-0864-x PMC2628981

[40] Percac‐Lima S , Lopez L , Ashburner JM , Green AR , Atlas SJ . The longitudinal impact of patient navigation on equity in colorectal cancer screening in a large primary care network. Cancer. 2014;120:2025‐2031. 24691564 10.1002/cncr.28682

[41] Ruggeri CE , Reed RE , Coyle B , Stoltzfus J , Fioravanti G , Tehrani R . Closing the gap: a resident‐led quality improvement project to improve colorectal cancer screening in primary care community clinics. J Grad Med Educ. 2020;12:1‐108. 32089801 10.4300/JGME-D-19-00144.1 PMC7012507

[42] Temucin E , Nahcivan NO . The effects of the nurse navigation program in promoting colorectal cancer screening behaviors: a randomized controlled trial. J Cancer Educ. 2020;35:112‐124. 30470978 10.1007/s13187-018-1448-z

[43] Walsh JM , Salazar R , Nguyen TT , et al. Healthy colon, healthy life: a novel colorectal cancer screening intervention. Am J Prev Med. 2010;39:1‐14. 20547275 10.1016/j.amepre.2010.02.020 PMC4282133

[44] Burhansstipanov L , Dignan MB , Schumacher A , Krebs LU , Alfonsi G , Apodaca CC . Breast screening navigator programs within three settings that assist underserved women. J Cancer Educ. 2010;25:247‐252. 20300914 10.1007/s13187-010-0071-4 PMC3544404

[45] Davis TC , Rademaker A , Bennett CL , et al. Improving mammography screening among the medically underserved. J Gen Intern Med. 2014;29:628‐635. 24366401 10.1007/s11606-013-2743-3 PMC3965756

[46] Davis TC , Arnold CL , Bennett CL , Wolf MS , Liu D , Rademaker A . Sustaining mammography screening among the medically underserved: a follow‐up evaluation. J Womens Health (Larchmt). 2015;24:291‐298. 25692910 10.1089/jwh.2014.4967 PMC4394885

[47] Han HR , Lee H , Kim MT , Kim KB . Tailored lay health worker intervention improves breast cancer screening outcomes in non‐adherent Korean‐American women. Health Educ Res. 2009;24:318‐329. 18463411 10.1093/her/cyn021 PMC2654061

[48] Highfield L , Rajan SS , Valerio MA , Walton G , Fernandez ME , Bartholomew LK . A non‐randomized controlled stepped wedge trial to evaluate the effectiveness of a multi‐level mammography intervention in improving appointment adherence in underserved women. Implement Sci. 2015;10:143. 26464110 10.1186/s13012-015-0334-x PMC4604615

[49] Margulies IG , Zwillenberg J , Chadda A , et al. Monitoring and developing a volunteer patient navigation intervention to improve mammography compliance in a safety net hospital. J Oncol Pract. 2019;15:e389‐e398. 30908139 10.1200/JOP.18.00424

[50] Marshall JK , Mbah OM , Ford JG , et al. Effect of patient navigation on breast cancer screening among African American Medicare beneficiaries: a randomized controlled trial. J Gen Intern Med. 2016;31:68‐76. 26259762 10.1007/s11606-015-3484-2 PMC4700012

[51] Molina Y , Kim SJ , Berrios N , et al. Patient navigation improves subsequent breast cancer screening after a noncancerous result: evidence from the patient navigation in medically underserved areas study. J Womens Health (Larchmt). 2018;27:317‐323. 28933653 10.1089/jwh.2016.6120 PMC5865251

[52] Phillips CE , Rothstein JD , Beaver K , Sherman BJ , Freund KM , Battaglia TA . Patient navigation to increase mammography screening among inner city women. J Gen Intern Med. 2011;26:123‐129. 20931294 10.1007/s11606-010-1527-2 PMC3019333

[53] Taplin SH , Barlow WE , Ludman E , et al. Testing reminder and motivational telephone calls to increase screening mammography: a randomized study. J Natl Cancer Inst. 2000;92:233‐242. 10655440 10.1093/jnci/92.3.233

[54] Corkrey R , Parkinson L , Bates L . Pressing the key pad: trial of a novel approach to health promotion advice. Prev Med. 2005;41:657‐666. 15917066 10.1016/j.ypmed.2004.12.008

[55] Hewett PC , Nalubamba M , Bozzani F , et al. Randomized evaluation and cost‐effectiveness of HIV and sexual and reproductive health service referral and linkage models in Zambia. BMC Public Health. 2016;16:785. 27519185 10.1186/s12889-016-3450-x PMC4983050

[56] Kitchener H , Gittins M , Rivero‐Arias O , et al. A cluster randomised trial of strategies to increase cervical screening uptake at first invitation (STRATEGIC). Health Technol Assess. 2016;20:1‐138. 10.3310/hta20680 PMC5046076 27632816

[57] Kitchener H , Gittins M , Cruickshank M , et al. A cluster randomized trial of strategies to increase uptake amongst young women invited for their first cervical screen: The STRATEGIC trial. J Med Screen. 2018;25:88‐98. 28530513 10.1177/0969141317696518 PMC5956569

[58] Taylor VM , Hislop TG , Jackson JC , et al. A randomized controlled trial of interventions to promote cervical cancer screening among Chinese women in North America. J Natl Cancer Inst. 2002;94:670‐677. 11983755 10.1093/jnci/94.9.670 PMC1592333

[59] Falk D , Cubbin C , Jones B , Carrillo‐Kappus K , Crocker A , Rice C . Increasing breast and cervical cancer screening in rural and border Texas with Friend to Friend plus patient navigation. J Cancer Educ. 2018;33:798‐805. 27900660 10.1007/s13187-016-1147-6 PMC10164719

[60] Lee BY , Jo HS . Evaluation of a navigator program for cancer screening of women in Korean communities. Asian Pac J Cancer Prev. 2011;12:271‐275. 21517270

[61] Beach ML , Flood AB , Robinson CM , et al. Can language‐concordant prevention care managers improve cancer screening rates? Cancer Epidemiol Biomarkers Prev. 2007;16:2058‐2064. 17932353 10.1158/1055-9965.EPI-07-0373

[62] Braun KL , Thomas WL Jr , Domingo JL , et al. Reducing cancer screening disparities in Medicare beneficiaries through cancer patient navigation. J Am Geriatr Soc. 2015;63:365‐370. 25640884 10.1111/jgs.13192 PMC4850231

[63] Dietrich AJ , Tobin JN , Cassells A , et al. Translation of an efficacious cancer‐screening intervention to women enrolled in a Medicaid managed care organization. Ann Fam Med. 2007;5:320‐327. 17664498 10.1370/afm.701 PMC1934974

[64] Percac‐Lima S , Ashburner JM , Zai AH , et al. Patient navigation for comprehensive cancer screening in high‐risk patients using a population‐based health information technology system: a randomized clinical trial. JAMA Intern Med. 2016;176:930‐937. 27273602 10.1001/jamainternmed.2016.0841

[65] The Community Guide . Cancer Screening: Patient Navigation Services to Increase Breast, Cervical, and Colorectal Cancer Screenings and Advance Health Equity. The Community Guide; 2023.

[66] Hoffmann TC , Glasziou PP , Boutron I , et al. Better reporting of interventions: template for intervention description and replication (TIDieR) checklist and guide. BMJ. 2014;348:g1687. 24609605 10.1136/bmj.g1687

[67] Battaglia TA , Fleisher L , Dwyer AJ , et al. National Navigation Roundtable Evidence‐Based Task Group. Barriers and opportunities to measuring oncology patient navigation impact: results from the National Navigation Roundtable survey. Cancer. 2022;128(Suppl 13):2568‐2577. 35699612 10.1002/cncr.33805

[68] Ustjanauskas AE , Bredice M , Nuhaily S , Kath L , Wells KJ . Training in patient navigation: a review of the research literature. Health Promot Pract. 2016;17:373‐381. 26656600 10.1177/1524839915616362 PMC4899310

[69] Mboineki JF , Wang P , Chen C . Fundamental elements in training patient navigators and their involvement in promoting public cervical cancer screening knowledge and practices: a systematic review. Cancer Control. 2021;28:10732748211026670. 34169777 10.1177/10732748211026670 PMC8236772

[70] Valverde PA , Burhansstipanov L , Patierno S , et al. Findings from the National Navigation Roundtable: a call for competency‐based patient navigation training. Cancer. 2019;125(24):4350‐4359. 31503340 10.1002/cncr.32470

[71] Lostao L , Joiner TE , Pettit JW , Chorot P , Sandín B . Health beliefs and illness attitudes as predictors of breast cancer screening attendance. Eur J Public Health. 2001;11:274‐279. 11582606 10.1093/eurpub/11.3.274

[72] Waller J , Robb K , Stubbings S , et al. Awareness of cancer symptoms and anticipated help seeking among ethnic minority groups in England. Br J Cancer. 2009;101(Suppl 2):S24‐S30. 19956159 10.1038/sj.bjc.6605387 PMC2790709

[73] Oncology Nursing Society , Association of Oncology Social Work , National Association of Social Workers . Oncology Nursing Society, The Association of Oncology Social Work, and the National Association of Social Workers joint position on the role of oncology nursing and oncology social work in patient navigation. Oncol Nurs Forum. 2010;37:251‐252. 20439209

[74] Budde H , Williams GA , Scarpetti G , Kroezen M , Maier CB . What are patient navigators and how can they improve integration of care? (Policy Brief, No. 44). European Observatory on Health Systems and Policies; 2022 https://www.ncbi.nlm.nih.gov/books/NBK577640/ 35129934

[75] Dalton M , Holzman E , Erwin E , et al. Patient navigation services for cancer care in low‐and middle‐income countries: a scoping review. PLoS ONE. 2019;14:e0223537. 31622363 10.1371/journal.pone.0223537 PMC6797131

